# Fructus gardeniae ameliorates anxiety-like behaviors induced by sleep deprivation via regulating hippocampal metabolomics and gut microbiota

**DOI:** 10.3389/fcimb.2023.1167312

**Published:** 2023-06-12

**Authors:** Dong Liu, Qianfei Wang, Ying Li, Zhenshuang Yuan, Zhiliang Liu, Junli Guo, Xin Li, Weichao Zhang, Yulei Tao, Jianqiang Mei

**Affiliations:** ^1^ Department of Emergency, The First Affiliated Hospital of Hebei University of Chinese Medicine, Shijiazhuang, Hebei, China; ^2^ Department of Traditional Chinese Medicine, Hebei General Hospital, Shijiazhuang, Hebei, China; ^3^ Department of Pharmacy, The First Affiliated Hospital, and College of Clinical Medicine of Henan University of Science and Technology, Luoyang, China; ^4^ School of Chinese Materia Medica, Beijing University of Chinese Medicine, Beijing, China; ^5^ Department of Emergency, Hebei Yiling Hospital, Shijiazhang, Hebei, China

**Keywords:** fructus *gardeniae*, sleep deprivation, anxiety, inflammation, hippocampal metabolomics, gut microbiota

## Abstract

Fructus *gardeniae* (FG) is a traditional Chinese medicine and health food for thousands of years of application throughout Chinese history and is still widely used in clinical Chinese medicine. FG has a beneficial impact on anxiety, depression, insomnia, and psychiatric disorders; however, its mechanism of action requires further investigation. This study aimed to investigate the effects and mechanisms of FG on sleep deprivation (SD)-induced anxiety-like behavior in rats. A model of SD-induced anxiety-like behavior in rats was established by intraperitoneal injection of p-chlorophenylalanine (PCPA). This was accompanied by neuroinflammation and metabolic abnormalities in the hippocampus and disturbance of intestinal microbiota. However reduced SD-induced anxiety-like behavior and decreased levels of pro-inflammatory cytokines including TNF-α and IL-1β were observed in the hippocampus of rats after 7 days of FG intervention. In addition, metabolomic analysis demonstrated that FG was able to modulate levels of phosphatidylserine 18, Phosphatidylinositol 18, sn-glycero-3-phosphocholine, deoxyguanylic acid, xylose, betaine and other metabolites in the hippocampus. The main metabolic pathways of hippocampal metabolites after FG intervention involve carbon metabolism, glycolysis/gluconeogenesis, pentose phosphate, and glycerophospholipid metabolism. 16S rRNA sequencing illustrated that FG ameliorated the dysbiosis of gut microbiota in anxious rats, mainly increased the abundance of Muribaculaceae and Lactobacillus, and decreased the abundance of Lachnospiraceae_NK4A136_group. In addition, the correlation analysis demonstrated that there was a close relationship between hippocampal metabolites and intestinal microbiota. In conclusion, FG improved the anxiety behavior and inhibited of neuroinflammation in sleep-deprived rats, and the mechanism may be related to the FG regulation of hippocampal metabolites and intestinal microflora composition.

## 1 Introduction

Sleep is an important physiological process that promotes the metabolism of the central nervous system, consolidates memory, and regulates mood (Xie et al., 2013; Nabaee et al., 2018). With the increasing demands for modern life and people’s external pressure, sleep time and quality has declined rapidly across societies. Sleep deprivation (SD) is a common disease that results in the reduction of people’s rest time and quality due to environmental or personal factors (Gou et al., 2017). Studies have found that SD leads to emotional abnormalities, learning and memory impairments, and poor immune function, along with the finding that severe sleep deprivation shortens the life span of animals (Faraut et al., 2012; Cao et al., 2020). Both acute and chronic sleep deprivation cause anxiety-like behaviors in animals (Manchanda et al., 2018; Liu et al., 2022). Research found that inflammation may be involved in the pathological process of anxiogenic-like behavior under sleep deprivation (Chen X. et al., 2019). Increased pro-inflammatory cytokines, decreased anti-inflammatory factors, and activation of microglia in the hippocampus have been found to play an important role in SD-induced anxiety behavior in rats (Wadhwa et al., 2018). Currently, the treatment of anxiety disorders is based on sedative-hypnotic drugs, however, some people suffer from adverse effects such as tolerance, addiction, and withdrawal symptoms. A recent study found that *Poria cocos* aqueous extract reduced inflammatory factors through the TNF-α/NF-κB signaling pathway and thereby improved SD-induced anxiety-like behavior (Zhang et al., 2022a). This provides the idea for the treatment of anxiety with traditional Chinese medicine.

Fructus *gardeniae* (FG) is derived from the dried and ripe fruit of a Rubiaceae plant, which is recorded in *Shennong Materia Medica*, the earliest Chinese work on traditional Chinese medicine. FG is an edible and medicinal plant with dual purposes. It has been reported to be effective at *clearing heat*, *purging fire*, *cooling the blood*, as well as *relieving toxins*, and *treating restlessness, irritability and upset.* The symptoms of restlessness in TCM are roughly consistent with mental illnesses such as anxiety and insomnia in Western medicine (Zhang et al., 2022b). The main components of FG include *iridoid glycosides, crocin, organic acids*, and *volatile oils*, which function in neuroprotective, antioxidant, antidepressant, and anti-inflammatory effects (Tian et al., 2022). FG exerts rapid antidepressant effects by increasing brain-derived neurotrophic factor (BDNF) in the hippocampus, and is the main component of the antidepressant effect of the Chinese medicine compound *Yueju* pill (Zhang et al., 2015). The active ingredient of FG is capable of reducing the activation of NF-*k*B and microglia in the central nervous system and playing an anti-inflammatory role (Wang et al., 2012). In this study, we evaluated the effect of FG on inflammatory factors in the brain of SD induced anxiety rats.

As sequencing and metabolomic technologies have advanced, they have been increasingly applied to medicine. Metabolomics uses analytical techniques based on liquid chromatography/mass spectrometry (LC/MS), gas chromatography/mass spectrometry (GC/MS), and nuclear magnetic resonance (NMR) spectroscopy to qualitatively and quantitatively analyze metabolites from living organisms and to search for the intrinsic links between metabolites and physiopathological changes at macroscopic and metabolic levels (Nicholson et al., 1999). The holistic and dynamic research model of metabolomics is aligned with the *holistic view* and *syndrome differentiation* of TCM, and can objectively and sensitively analyze the effects of TCM on metabolites from living organisms (Han et al., 2020). Si et al. found that *Lilium brownii* interfered with the metabolic pathways of arachidonic acid metabolism and 5-hydroxytryptaminergic synapses in the hypothalamus and hippocampus to normalize the metabolic phenotype of insomnia in rats based on LC-MS/MS-based metabolomics (Si et al., 2022). In this study, the UPLC-Q-Exactive-Orbitrap-MS technology has been used to detect hippocampal metabolites during SD-induced anxiety behavior in rats and to evaluate the mechanism of drugs.

The “brain-gut axis” plays an important role in regulating sleep and mood. There is a bidirectional link between the intestinal microbiota and the central nervous system, with intestinal bacteria influencing brain function, sleep structure, and mood stability through neurological, endocrine, and immune signals (Collins et al., 2012). Similarly, the central nervous system can also influence intestinal permeability and the environment of intestinal microbiota through the three above pathways, and alter the composition of the gut microbiota (Bauer et al., 2016). Studies have found that gut microbiome play a bridging role in immune function and mental-emotional disorders (Sundman et al., 2017). Chronic sleep deprivation induces anxiety-like behavior, inflammatory responses, as well as neurotransmitter and gut dysfunction (Zhang et al., 2022a). Wang, et al., found that transplantation of SD microbiota into germ-free mice resulted in a significant increase in activation of inflammatory factors TNF-α, IL-1β, IL-6 and microglia in the hippocampus and prefrontal cortex, accompanied by cognitive dysfunction (Wang et al., 2021). This suggests that gut microbiome plays an important role in abnormal mental behaviors and responses such as inflammation, anxiety and cognition in hosts.

Based on the above studies, in this study, we tested the effects of FG in ameliorating anxiety and suppressing hippocampal inflammation by establishing an SD-induced anxiety model. To clarify the anxiolytic mechanism of FG, the effects on hippocampal metabolites and intestinal microbiota of rats in the anxiety model were evaluated by combining UPLC-Q-Exactive-Orbitrap-MS and 16S rRNA sequencing techniques. Finally, the correlation analysis of hippocampal differential metabolites and intestinal differential microbiome was performed using Spearman’s rank correlation coefficient. It is hypothesized that FG improves anxiety-like behavior and inhibit neuroinflammatory factors, and the mechanism is related to the intervention of hippocampal metabolites and the increase of beneficial bacteria in intestinal microbiome.

## 2 Materials and methods

### 2.1 Drugs, reagents, and preparation

The Chinese medicine FG (Lot No. 21102882 Shijiazhuang City, Hebei Province Shenwei Pharmaceutical Co., Ltd.) was provided by the Chinese pharmacy of Hebei General Hospital. FG granules were added to water at 100°C and stirred to fully dissolve into FG low dose (1.05g/kg/d) and FG high dose (4.15g/kg/d) respectively. Hippocampal samples detected by enzyme-linked immunosorbent assay (ELISA) kits for TNF-α (L220408138 Uscn Life Science, Inc, Wuhan, China) and IL-1β (L220524789 Uscn Life Science, Inc, Wuhan, China).PCPA (Lot No. 7GFOE-DR TCI (Shanghai) Chemical Industry Development Co., Ltd.) was dissolved in weak alkaline saline (ph: 7-8). The dissolution of PCPA can be promoted by adding proper amount of sodium bicarbonate into the saline to prepare a weak alkaline physiological saline. Estazolam (Lot No. H37023047 Shandong Xinyi Pharmaceutical Company) were prepared with water to a dose of 0.5 mg/kg/d. Estazolam, which is a positive control drug, has been in clinical for a long time and is one of the standard drug for the treatment of anxiety and insomnia (Xu et al., 2018).

### 2.2 Animals and treatments

Forty male SD rats (5 weeks old, 151-175g) were purchased from Beijing Vital River Laboratory Animal Technology Co Ltd (license number SCXK (Beijing) 2021-0011). Animals were housed at the Experimental Animal Center of the Hebei University of Chinese Medicine (Shijiazhuang, China). The animals are kept in a clean laboratory environment for 12 h at alternating day and night with an ambient temperature of (23 ± 2) °C and humidity of 50% ± 5%, with free access to water and food. The experiment was approved by the Ethics Committee of Hebei university of Chinese Medicine (No. DWLL202212018).

**Figure 1:**
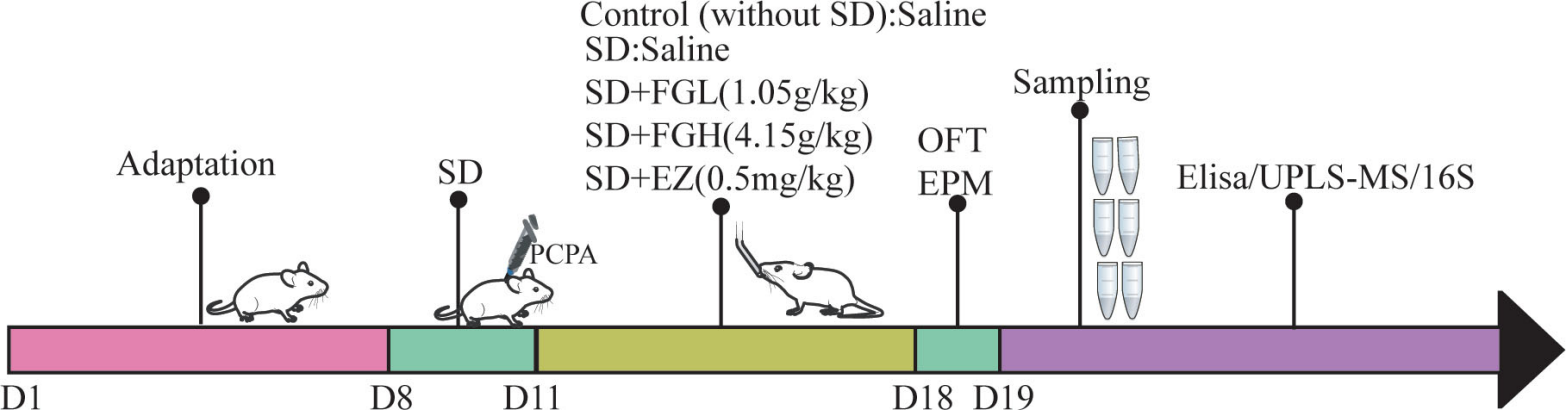
Schematic illustration of anxiety-like behavior induced by sleep deprivation in rats. After one week of adaptation, except for the blank group, the other groups successfully established sleep deprivation rat models by injecting PCPA at 9:00-10:00 am for three consecutive days. After 7 days of continuous gavage, anxiety was evaluated by behavioral tests (OFT and EPM). After anesthesia and execution, hippocampus and feces were taken for Elisa, UPLS-MS and 16S sequencing respectively. SD, sleep deprivation; PCPA, p-chlorophenylalanine; FGL, Fructus *gardeniae* low (1.05g/kg); FGH, Fructus *gardeniae* high (4.15g/Kg); EZ, Estazolam (0.5mg/kg); EPM, Elevated plus maze; OFT, Open field test; Elisa, Enzyme-linked immunosorbent assay.

After one week of training, SD rats were randomly divided into the following five groups (*n* = 8): control group, sleep deprivation group (SD), SD + low dose FGL group (SD + FGL), SD + high dose group (SD + FGH) and SD + estazolam group (SD + EZ). As shown in (**Figure 1**), In addition to the control group, which was given an equal volume of saline, other groups were given PCPA, which was injected intraperitoneally at 400mg·kg^- 1^ in a volume of 10mL·kg^-1^ once a day from 9:00 to 10:00 am for 3 days. Compared to the control group, these rats gradually showed restlessness, increased aggressiveness, and circadian rhythm disturbance during the modeling process, indicating the initial success of modeling. The rats in the intervention group were given the interventional drugs by gavage (10 mL·kg^- 1^) per day for 7 days. The rats in the normal group and the model group were given an equal volume of pure water once a day for 7 days.

### 2.3 Animal behavior test

#### 2.3.1 Open field test (OFT)

The OFT is conducted in a quiet, low-light environment and is used to assess the level of spontaneous anxiety in rodents. The time spent by the rats in the central area and the frequency of entering the central area within 5 minutes were recorded by the animal behaviour analysis system (Beijing Shuolingyuan Technology Co., Ltd). At the end of the test, the open field was wiped with 75% alcohol to remove feces and urine to avoid the odor left behind impacting the results of the next test.

#### 2.3.2 Elevated plus maze (EPM)

The EPM was designed to measure the anxiety of the animals by taking advantage of the natural curiosity of rats and their tendency to darkness association. Behavioral software counted the following indicators in 5 minutes: the number of rats entering and the residence time in both open and closed arms. We calculated the percentage of open arm entries (OE% = open arm entries/total entries in both arms × 100%) and percentage of open arm times (OT% = open arm times/total time in both arms × 100%). Before allowing the next animal into the EPM, the odor of the maze was removed with 75% alcohol, and the feces and urine of the rats were also removed. If the rat accidentally fell from the maze during the experiment, the data of that experiment were not included in the statistics.

### 2.4 Samples collection and preparation

The rats were fasted for 12 hours before intraperitoneal anesthesia with sodium pentobarbital, decapitated after blood was drawn from the abdominal aorta and the brains rapidly dissected on ice. Hippocampal tissues were obtained and quickly frozen in liquid nitrogen and stored at -80 °C in the freezer for ELISA and metabolomic assays. The intestinal tube was severed with sterile scissors, and fresh fecal samples were collected from each rat, placed into 2 ml sterile tubes, and stored in a -80 °C freezer until DNA extraction was performed for gut microbiota sequencing analysis.

### 2.5 ELISA

Hippocampal tissue was thawed in a refrigerator from -80°C, accurately weighed and mixed with 0.9% normal saline to a weight/volume ratio of 1:9. The tissue was then ground on ice to prepare 10% homogenate. The homogenate was then centrifuged with 3000 rpm at 4°C for 10 minutes and the supernatant was obtained for ELISA.

### 2.6 Non-target metabolomics

#### 2.6.1 Pre-sampling of brain hippocampal samples

Frozen tissue samples were removed from the -80°C freezer and thawed at room temperature. 50 mg of each sample was weighed and 200 μL of precooled water and 800μL of precooled methanol/ethylene proline (1:1, v/v) were added and mixed evenly. This mixture was ultrasonicated in an ice bath for 1h, and it was left standing at - 20°C for 2h. The supernatant was centrifuged at a high speed of 13000 rpm at 4°C for 15min. The supernatant was dried in a high-speed vacuum centrifuge and redissolved in 100 μL of a methanol-water solution (1:1, V/V). The supernatant was centrifuged at 20000 rpm for 20 min at 4°C and analyzed by LC-MS. 10mL of supernatant was drawn from each sample and placed in a centrifuge tube. Quality control (QC) samples were prepared 1 min after vortex mixing to assess the stability of the test instrument and experimental results.

#### 2.6.2 LC-MS Analysis

2.6.2.1 Chromatographic conditions

The analytical instrument used in this experiment was the Nexera x 2LC-30AD high-performance liquid chromatography system (Shimadzu, Japan). The column employed in this study was an ACQUITY UPLC HSS T3 (2.1 × 150 mm, 1.8 μm) with a column temperature of 40°C, sample volume of 6 μL, and a flow rate of 0.3 mL/min. Chromatographic mobile phases: A: 0.1% formic acid aqueous solution, B: acetonitrile; chromatographic gradient elution procedure as follows: 0% B at 0-2 min; 0-48% B at 2-6 min; 48-100% B at 6-10 min; 100% B at 10-12.1 min; and 100-0% B at 12.1-15 min.

##### 2.6.2.2 Mass spectroscopy conditions

The analytical instrument used in the mass spectrometry experiment is the Q-Exactive Plus (Thermo Scientific, San Jose, USA), provided by Bioprofile Biotech Ltd. (Shanghai, China). Data were collected in positive and negative ion modes using a high-resolution electrospray ionization (HESI) source. The ionization conditions are as follows: spray voltage: 3.8kV (+) and 3.2kV (-); Capillary Temperature:320 (±); Sheath Gas:30 (±); Aux Gas:5 (±); Probe Heater Temp: 350 (±);S-LensRF level:5. Mass spectrum acquisition time was 15min, parent ion scanning range 80-1200 *m/z*, primary mass spectrum resolution was 70000 m/z 200, AGC target: 3e6, Level 1 Maximum IT:100 ms. Secondary mass spectrometry acquisition: after each full scan, triggering and acquisition of secondary mass spectrograms from 10 parent ions with the highest intensity was performed. Secondary mass spectrometry resolution: 17500 m/z 200, AGC target:1e5, secondary Maximum IT:50 ms, MS2 Activation Type: HCD, Isolation window: 2 m/z, Normalized collision energy (Stepped):20, 30,40.

#### 2.6.3 Data pre-processing

Peak alignment, extraction, and matching were all performed on the raw data using MSDIAL software to obtain peak list information including retention time and chromatographic peak area. For the extracted data, the ion peaks with missing values > 50% in the group were deleted and did not participate in subsequent statistical analyses. The positive and negative ion data were normalized by the total peak area. The positive and negative ion peaks were integrated and R software was used for pattern recognition. After data were pretreated by unit variance scaling (UV), subsequent data analysis was performed.

#### 2.6.4 Multivariate statistical analysis and identification of differential metabolites

Preprocessed data were imported into R software. First, unsupervised principal component analysis (PCA) was performed to observe if there were abnormal data. Next, a supervised orthogonal partial least square-discriminant analysis (OPLS-DA) was performed, and 200 rounds of permutation verification were performed. The intercept of R^2^ and Q^2^ was used to judge whether the data were fitted appropriately, so as to determine the reliability and stability of the model. The significance analysis for each substance was obtained by univariate statistical analysis, and compounds satisfying both the conditions of *p* < 0.05 and VIP > 1 were considered as significantly different compounds. Through matching and comparison with network open source databases including the Human Metabolome Database (HMDB, http://www.hmdb.ca/), Metlin, and local databases, differential compounds were identified. The obtained differential metabolites were input into the the Kyoto Encyclopedia of Genes and Genomes (KEGG, http://www.genome.jp/kegg/) database for enrichment analysis and the metabolic pathways of the identified differential markers were constructed to investigate their biological significance.

### 2.7 16S rRNA sequencing and microbial community analysis

#### 2.7.1 Extraction and detection of total DNA

DNA extraction from fecal samples was performed according to OMEGA Soil DNA Kit (M5635-02) (Omega Bio-Tek, Norcross, Ga., USA) instructions, and the extracted DNA was quantitatively analyzed using 0.8% agarose gel electrophoresis and a UV spectrophotometer (A260/280).

#### 2.7.2 Target sequence amplification and sequencing

The fecal 16S rRNA V3-V4 region DNA fragment was PCR-amplified using barcode-indexed primers 338F (5`-ACTCCTACGGGAGGCAGCA-3`) and 806R (5` -GGACTACHVGGGTWTCTAAT-3`). Amplification conditions were as follows: a pre-denaturation temperature of 98°C was set for 5 min, 25 cycles were run: 98°C denaturation for 30s, 53°C annealing for 30s, 72°C extension for 45s, and finally a 72°C extension for 5 min. PCR amplicons were purified using Vazyme V AHTSTM DNA Clean Beads (Vazym, Nanjing, China) and quantified using the Quant iT PicoGreen dsDNA detection kit (Invitrogen, Carlsbad, CA,USA). After a separate quantification step, amplicons were pooled in equal amounts and the terminal 2×250 regions were sequenced using the Illumina NovaSeq platform and the Novasek 6000SP kit (500 cycles).

#### 2.7.3 Bioinformatics analysis of 16s rRNA

This study used QIIME2 version 2019.4 which based on the official teaching procedure (https://docs.qiime2.org/2019.4/tutorials/) performs quality control, denoising, chimera removal, and other processing on original sequence data to generate operational taxonomic units (OTU). Alpha diversity analyzes the species diversity and complexity of a sample, including seven indicators: CHAO1 index, observed species index, Shannon index, Simpson index, Faith’s PD index, and Pielou’s evenness index. The UNIFRAC distance metric was used for beta diversity analysis, and differences in sample species complexity were assessed by principal coordinate analysis (PCA) and non-metric multidimensional scaling (NMDS). Based upon the taxonomic information, composition and abundance tables of the samples at the taxonomic levels of phylum, class, order, family, genus, and species were obtained. Permanova (Adonis) software analysis was used to evaluate the difference between microbial community structure among the groups. Linear discriminant analysis (LDA) and effect size (LEfSe) were used to screen different groups of microbial community markers.

### 2.8 Data statistical analysis

Behavioral data and inflammatory marker data were subjected to one-way ANOVA using GraphPad Prism 8 and expressed as mean ± standard error. *p* < 0.05, *p* < 0.01 and *p* < 0.001 indicated a significant difference. Spearman’s correlation analysis was used to analyze the association between different microbiota and metabolites.

## 3 Results

### 3.1 Effects of FG on SD-induced anxiety-like behavior

**Figure 2:**
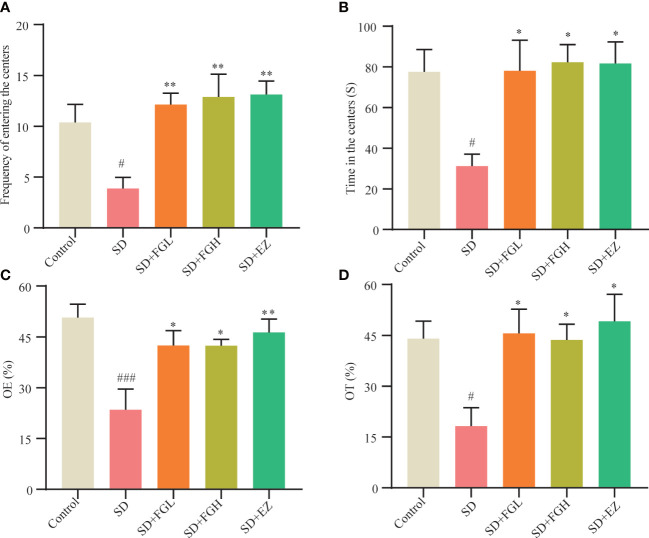
Effects of FG on anxiety behavior of sleep deprivation rats. Results of OFT in behavior test: **(A)** Frequency of entering the centers **(B)** Time in the centers. Results of EPM in behavior test: **(C)** open arm enters percentage (OE%). **(D)** open arm time percentage (OT%). The all groups differences were assessed using one-way ANOVA. The results are expressed as mean ± standard error. SD group vs control group: ^#^ *P*<0.05, ^###^ *P*<0.001. Treatment groups vs SD group:*P<0.05, **P<0.01.

The OFT was used to assess anxiety behavior, including time and frequency of rats entering the central area. As shown in (**Figures 2A, B**), Compared to the control group, the frequency (*p* < 0.05) and time (*p* < 0.05) in the central region of the SD group were significantly decreased. However, FGL and FGH increased both the frequency (*p* < 0.01) and time (*p* < 0.05) of rats entering the central region. The positive drug group EZ increased central zone frequency (*p* < 0.01) and time (*p* < 0.05) as well. The anxiety-like behavior of SD rats was evaluated by OE% and OT% in the EPM (**Figures 2C, D**). Compared to the control group, the SD group had significantly reduced OE% (*p* < 0.001) and OT% (*p* < 0.05). After FGL and FGH intervention, the OE% (*p* < 0.05) and OT% (*p* < 0.05) of rats increased significantly. The EZ group significantly increased the OE% (*p* < 0.01) and OT% (*p* < 0.05).

### 3.2 Effects of FG on hippocampal inflammation in rats

**Figure 3:**
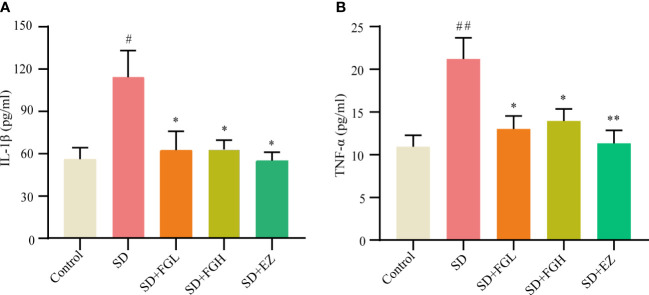
Concentration of inflammatory factors in hippocampus of SD-induced anxiety rats was detected by ELISA. **(A, B)** Hippocampal inflammatory factor IL-1β and TNF-α concentrations. SD group vs control group: ^#^ *p*<0.05, ^##^ *p*<0.01; Treatment groups vs SD group: *p<0.05, **p<0.01. Differences were assessed by one-way ANOVA. Values are presented as the means ± standard error.

After the behavioral experiment, we continued to explore the role of FG on inflammation in the hippocampus of SD rats. This study found that the SD group had significantly increased levels of IL-1β (*p* < 0.05) and TNF-α (*p* < 0.01) in the hippocampus of rats, as shown in (**Figure 3**). IL-1β (*P* < 0.05) and TNF-α (*p* < 0.05) were significantly decreased after 7 days of FG-L and FG-H treatment. The EZ group significantly decreased the levels of IL-1β (*p* < 0.05) and TNF-α (*p* < 0.01) in the hippocampus.

### 3.3 Non-targeted metabolomics analysis

#### 3.3.1 Methodological investigation

**Figure 4:**
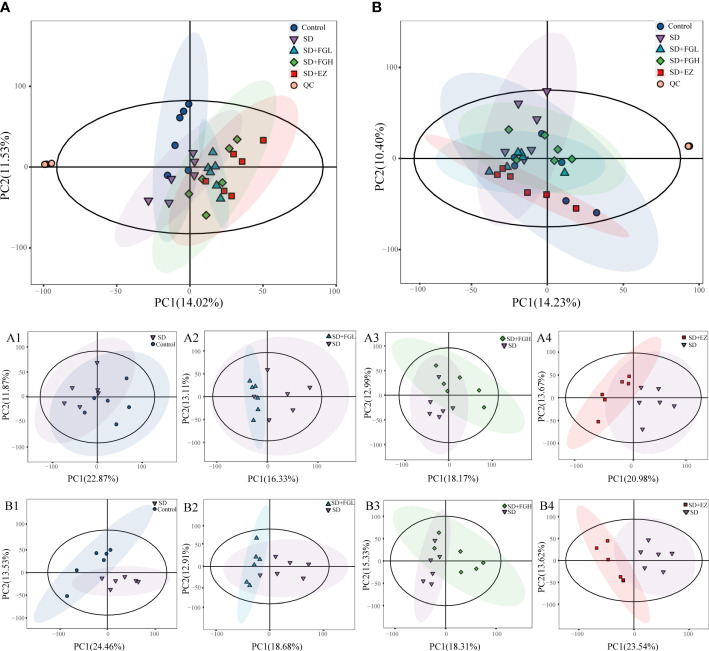
Results of PCA statistical analysis in hippocampus metabolomics. **(A)** PCA analysis results among five groups in positive mode. (A1-A4) PCA analysis between two groups in positive mode. (A1) Control vs SD (R^2^X:0.557, Q^2^:0.002). (A2) SD+FGL vs SD (R^2^X:0.536, Q^2^:-0.561). (A3) SD+FGH vs SD (R^2^X:0.550, Q^2^:-0.438). (A4) SD+EZ vs SD (R^2^X:0.565, Q^2^:-0.192); **(B)** PCA analysis results among five groups in negative mode. (B1-B4): PCA analysis profile between two groups in negative mode. (B1) Control vs SD (R^2^X:0.579, Q^2^:0.030). (B2) SD+FGL vs SD (R^2^X:0.540, Q^2^:-0.208). (B3) SD+FGH vs SD (R^2^X:0.552, Q^2^:-0.356). (B4) SD+EZ vs SD (R^2^X:0.579, Q^2^:0.119).

In this study, the peaks extracted from all the experimental samples and QC samples were UV-treated and then analyzed by PCA. After 7 cycles of cross-validation, the PCA model was obtained. As shown in (**Figures 4A, B**), QC samples in positive and negative ion mode are closely clustered, indicating the stability and reproducibility of this experiment.

#### 3.3.2 Metabolic profiling analysis

**Figure 5:**
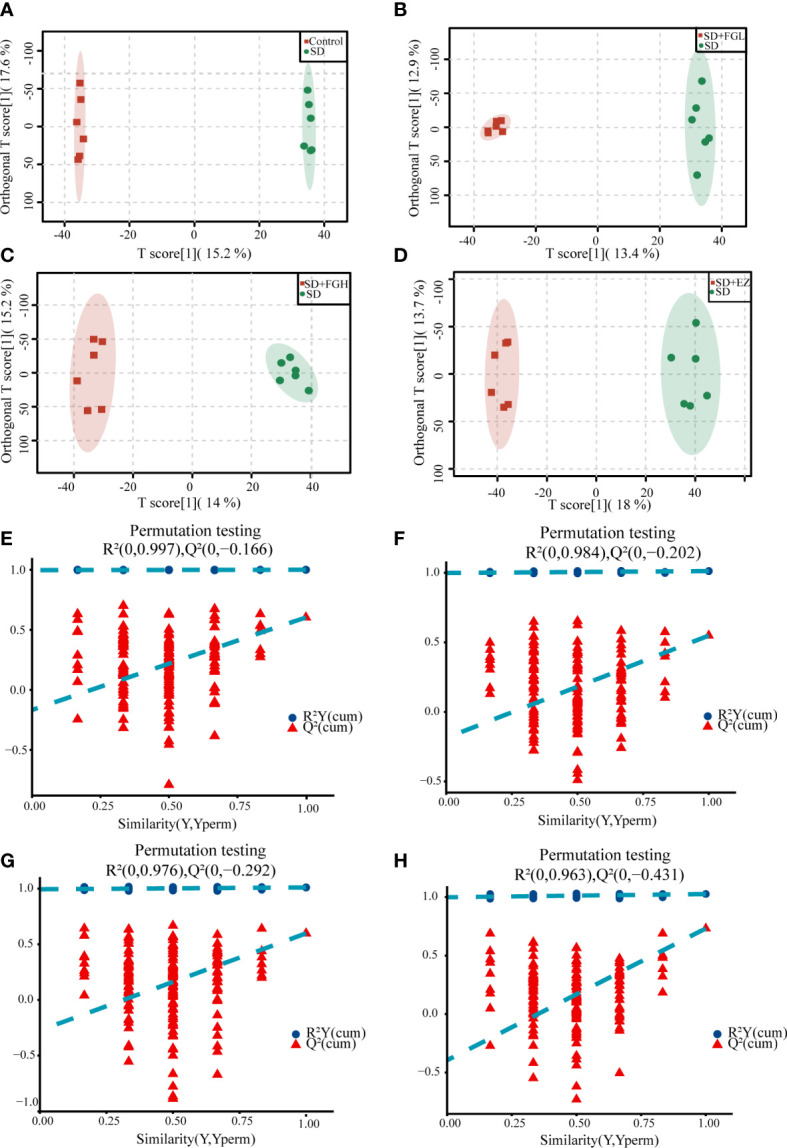
OPLS-DA analysis results and the model stability verification. **(A-D)** OPLS-DA analysis results in positive mode. **(A)** Control vs SD (R^2^Y=1.000, Q^2 = ^0.603). **(B)** SD+FGL vs SD (R^2^Y=0.997, Q^2 = ^0.534). **(C)** SD+FGH vs SD (R^2^Y=0.992, Q^2 = ^0.579). **(D)** SD+EZ vs SD (R^2^Y=0.991, Q^2 = ^0.696); **(E-H)** OPLS-DA model stability verification results in positive mode. The abscissa represents the correlation between Y in the random group and Y in the original group, and the ordinate represents the score of R^2^ and Q^2^.The Q^2^ and R^2^ values on the left are lower than the original point on the right, or the intercept of the Q^2^ regression line is negative. **(E)** Control vs SD. **(F)** SD+FGL vs SD. **(G)** SD+FGH vs SD. **(H)** SD+EZ vs SD.

To further determine the metabolic differences between the groups, metabolic data were analyzed by multivariate statistical methods. PCA is an unsupervised principal component analysis used to observe overall distribution trends between groups, as well as identify and eliminate discrete points. The PCA score plots for all groups and between the two groups in positive and negative ion mode are shown in **Figure 4**. Each point represents an experimental sample, and the greater the distance between the points of different groups, the greater the variability of the samples. OPLS-DA is a supervised principal component analysis for further differentiation of metabolite profiles and screening of potential marker metabolites. A clear separation between the two groups was visible in both positive ion mode (**Figures 5A-D**) and negative ion mode (**Supplementary Figures 1A-D**), indicating a significant alteration of the metabolic profile in the hippocampus of SD rats. R^2^Y and Q^2^ of the OPLS-DA model represent the interpretation rate and prediction rate, respectively. The closer the above two parameters are to 1, the more stable the model is. In order to verify the reliability of this model, 200 rounds of permutation tests were conducted under positive ion mode (**Figures 5E-H**) and negative ion mode (**Supplementary Figures 1E-H**), respectively. Upon visualization, the Q^2^ value on the left side was lower than the original point on the right side or the intercept of the Y axis was negative. The model did not have any issues being fit, which demonstrated that the OPLS-DA model had high reliability and strong predictability.

#### 3.3.3 Identification of differential metabolites in hippocampus

**Figure 6:**
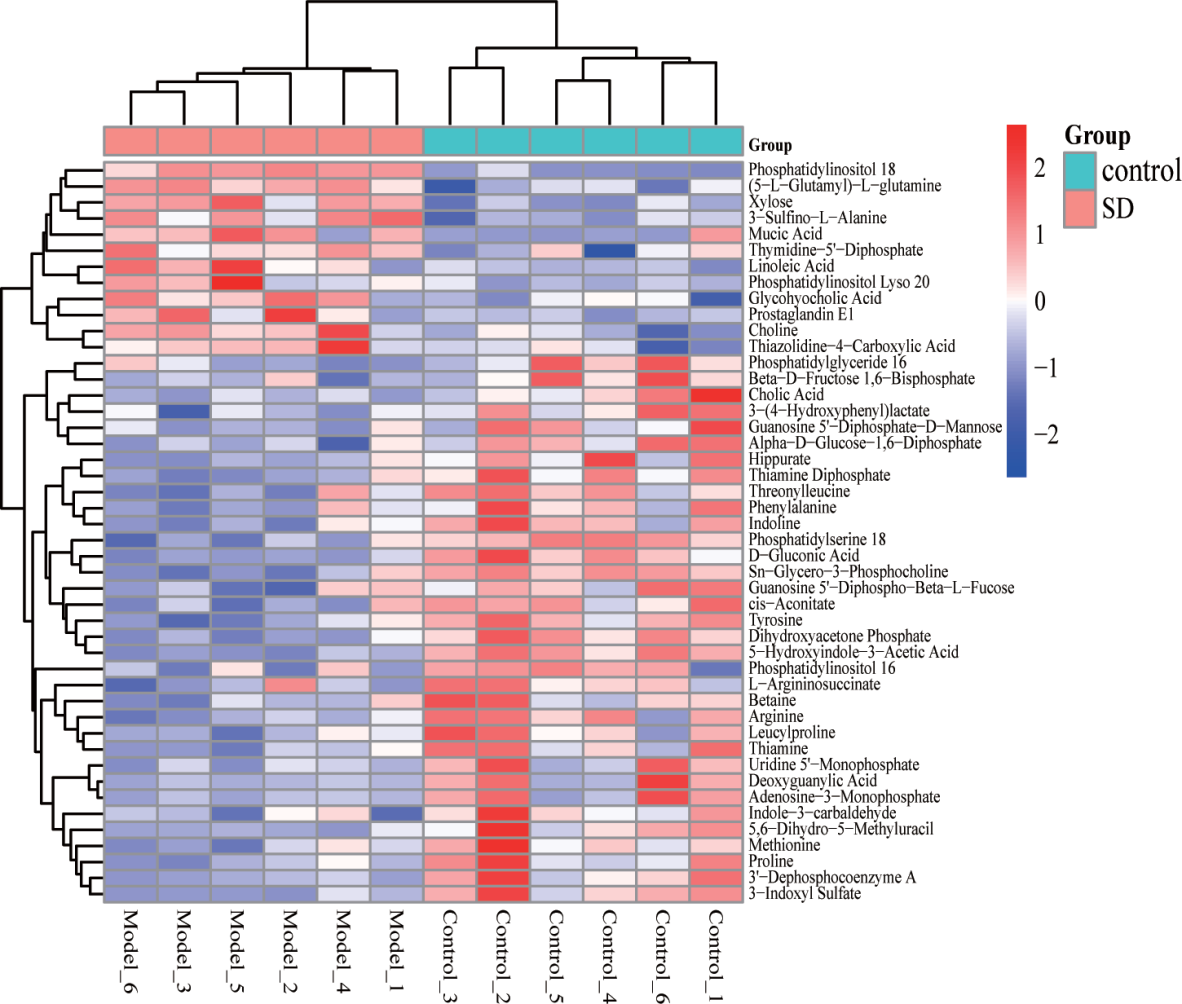
Heat map of different metabolite levels in hippocampus. The figure shows SD group vs control group. The abscissa represents the sample, and the ordinate represents the metabolites. Red and blue represent up-regulation and down-regulation of hippocampal metabolites, respectively.

The OPLS-DA model was used to screen different variables with VIP > 1 and *P* < 0.05 in each group. A total of 46 Hippocampal metabolites with significant differences were identified through reference to the HMDB database and metabolite secondary fragment ions (**Table 1**). Compared to the control group, the levels of 12 different metabolites in the SD group, including Phosphatidylinositol 18, Xylose, 3-Sulfino-L-Alanine, (5-L-Glutamyl)-L-glutamine, Choline, Mucic Acid, Linoleic Acid, and Glycohyocholic Acid were significantly increased. 34 metabolites such as tyrosine, d-gluconic acid, sn-glycero-3-phosphocholine, and dihydroxyacetone phosphate were significantly reduced, suggesting that these metabolites may be involved in SD-induced anxiety-like behavior in rats. However, after oral administration of FGL, 15 hippocampal metabolites including phosphatidylinositol 18, D-Gluconic Acid, betaine, adenosine-3-monophosphate, hippurate, deoxyguanylic acid, and alpha-D-glucose-1,6-diphosphate had approached to the levels of the control group. 14 hippocampal metabolites including xylose, choline, sn-glycero-3-phosphocholine, phosphatidylserine 18, adenosine-3-monophosphate, and deoxyguanylic acid were detected in levels comparable to the control group after oral administration of FG. 17 hippocampal metabolites including d-gluconic acid, xylose, sn-glycero-3-phosphocholine, phosphatidylserine 18, dihydroxyacetone phosphate, 3-sulfino-l-alanine, and 3-indoxyl sulfate were significantly regulated to control group levels in the EZ group. In order to better visualize the distribution and extent of the differential expression of hippocampal metabolites between the two groups, we drawn the volcanic map (**Supplementary Figure 2**) and the heat map (**Figure 6**, **Supplementary Figure 3**).

**Table 1:** Changes of metabolites in hippocampus of anxiety induced by sleep deprivation in rats.

NO	RT	Adduct	MetaboName	Caled m/z	Error	Formula	Trend			
							SD	SD+FGL	SD+FGH	SD+EZ
1	13.403	[M-H]-	Phosphatidylinositol 18	885.54987	-0.62	C47H83O13P	↑^###^	↓^***^	−	↓^***^
2	5.714	[M+H]+	5-Hydroxyindole-3-Acetic Acid	192.06552	-2.24	C10H9NO3	↓^###^	−	−	−
3	1.286	[M-H]-	D-Gluconic Acid	195.05103	-6.77	C6H12O7	↓^###^	↑^***^	−	↑^***^
4	1.268	[M]+	Sn-Glycero-3-Phosphocholine	258.10956	2.01	C8H21NO6P	↓^###^	↑^***^	↑^***^	↑^*^
5	1.435	[M+K]+	Xylose	189.01598	-1.69	C5H10O5	↑^###^	−	↓^*^	↓^***^
6	13.409	[M+Hac-H]-	Phosphatidylserine 18	834.52905	-0.80	C46H78NO10P	↓^###^	↑^***^	↑^***^	↑^***^
7	1.294	[M-H]-	Dihydroxyacetone Phosphate	168.99075	-9.70	C3H7O6P	↓^###^	↑^***^	−	↑^***^
8	5.852	[M-H]-	3-Indoxyl Sulfate	212.0023	-4.81	C8H7NO4S	↓^##^	−	−	↑^*^
9	4.018	[M+H]+	Tyrosine	182.08118	-0.49	C9H11NO3	↓^##^	−	−	−
10	1.372	[M-H2O-H]-	3-Sulfino-L-Alanine	152.002	-8.03	C3H7NO4S	↑^##^	−	−	↓^*^
11	1.361	[M-H]-	(5-L-Glutamyl)-L-glutamine	274.10446	-3.54	C10H17N3O6	↑^##^	−	↓^***^	−
12	5.124	[M-H]-	3'-Dephosphocoenzyme A	686.1416	-4.90	C21H35N7O13P2S	↓^##^	−	−	−
13	4.656	[M+K]+	Thiamine Diphosphate	448.04001	-1.85	C12H19N4O7P2S+	↓^##^	−	−	−
14	1.252	[M]+	Choline	104.10645	8.55	C5H14NO	↑^##^	−	↓^***^	−
15	1.234	[M+H]+	Arginine	175.11896	-3.77	C6H14N4O2	↓^##^	−	−	−
16	1.34	[M+H]+	5,6-Dihydro-5-Methyluracil	129.06586	0.31	C5H8N2O2	↓^##^	−	↑^*^	−
17	5.166	[M+H]+	Indoline	120.08077	0.75	C8H9N	↓^#^	−	−	−
18	1.449	[M-H]-	Alpha-D-Glucose-1,6-Diphosphate	338.98877	-1.27	C6H14O12P2	↓^#^	↑^***^	−	−
19	6.82	[M+H]+	Thiamine	304.07547	9.44	[C12H17N4OS]+	↓^#^	−	−	−
20	5.199	[M+H]+	Threonylleucine	233.151	-9.99	C10H20N2O4	↓^#^	−	−	−
21	5.86	[M-H]-	Hippurate	178.05	-1.68	C9H9NO3	↓^#^	↑^***^	↑^*^	↑^***^
22	1.375	[M+H]+	Proline	116.0706	1.29	C5H9NO2	↓^#^	−	−	−
23	5.442	[M-H]-	3-(4-Hydroxyphenyl)lactate	181.05063	-5.25	C9H10O4	↓^#^	−	−	−
24	2.277	[M+H]+	Methionine	150.058	1.73	C5H11NO2S	↓^#^	−	−	−
25	2.73	[M-H]-	Adenosine-3-Monophosphate	346.05582	-5.29	C10H14N5O7P	↓^#^	↑^***^	↑^***^	↑^***^
26	9.173	[M+H-2H2O]+	Glycohyocholic Acid	488.29901	1.62	C26H43NO6	↑^#^	−	−	−
27	2.731	[2M-H]-	Deoxyguanylic Acid	693.12292	-9.51	C10H14N5O7P	↓^#^	↑^**^	↑^***^	↑^*^
28	1.285	[M-H]-	Mucic Acid	209.03029	-5.69	C6H10O8	↑^#^	↓^*^	−	↓^*^
29	2.134	[M-H]-	Uridine 5'-Monophosphate	323.02859	-0.19	C9H13N2O9P	↓^#^	↑^*^	↑^***^	−
30	8.279	[M+NH4]+	Cholic Acid	426.32141	-4.93	C24H40O5	↓^#^	−	−	−
31	11.587	[M-H]-	Linoleic Acid	279.23294	-0.86	C18H32O2	↑^#^	↓^*^	−	↓^*^
32	1.401	[M+H]+	Thiazolidine-4-Carboxylic Acid	134.02702	-1.94	C4H7NO2S	↑^#^	−	↓^*^	−
33	5.328	[M+H]+	Indole-3-carbaldehyde	146.06004	-1.78	C9H7NO	↓^#^	−	−	−
34	1.631	[M+H]+	Thymidine-5'-Diphosphate	403.03021	-7.27	C10H16N2O11P2	↑^#^	−	↓^***^	−
35	13.403	[2M-H]-	Phosphatidylinositol Lyso 20	619.28888	-3.75	C29H49O12P	↑^#^	↓^***^	−	−
36	7.52	[M-H]-	Prostaglandin E1	353.23334	-2.18	C20H34O5	↑^#^	−	−	−
37	5.262	[M+H]+	Leucylproline	229.15511	-5.15	C11H20N2O3	↓^#^	−	−	−
38	13.399	[M-H]-	Phosphatidylglyceride 16	747.51819	2.29	C40H77O10P	↓^#^	−	↑^***^	−
39	1.304	[M+H]+	Betaine	118.08626	-0.34	C5H11NO2	↓^#^	↑^***^	−	−
40	5.166	[M+H]+	Phenylalanine	166.08626	-2.89	C9H11NO2	↓^#^	−	−	−
41	3.455	[M-H]-	Guanosine 5'-Diphospho-Beta-L-Fucose	588.07495	-2.81	C16H25N5O15P2	↓^#^	−	↑^***^	↑^***^
42	1.554	[M+H]+	Beta-D-Fructose 1,6-Bisphosphate	341.00333	-2.61	C6H14O12P2	↓^#^	↑^***^	−	−
43	2.166	[M-H]-	Guanosine 5'-Diphosphate-D-Mannose	604.06989	-2.23	C16H25N5O16P2	↓^#^	−	−	↑^***^
44	1.316	[M+H]+	L-Argininosuccinate	291.12991	-3.78	C10H18N4O6	↓^#^	−	−	−
45	13.404	[M+Hac-H]-	Phosphatidylinositol 16	857.51855	-4.34	C45H79O13P	↓^#^	−	−	↑^*^
46	2.609	[M-H]-	cis-Aconitate	173.00916	-8.67	C6H6O6	↓^##^	−	−	↑^*^

"↑" and "↓" indicate relative increasing or decreasing trends of metabolites, and "-" indicates no significant difference between the two groups. ^#^p<0.05, ^##^p<0.01, ^###^p<0.001 indicate statistically significant differences between the control group and the sleep deprivation (SD) group. *P<0.05, **p<0.01, ***p<0.001 indicate statistically significant differences between the treatment groups (SD+FGL/SD+FGH/SD+EZ) and the SD group.

#### 3.3.4 Analysis of metabolic pathways

**Figure 7:**
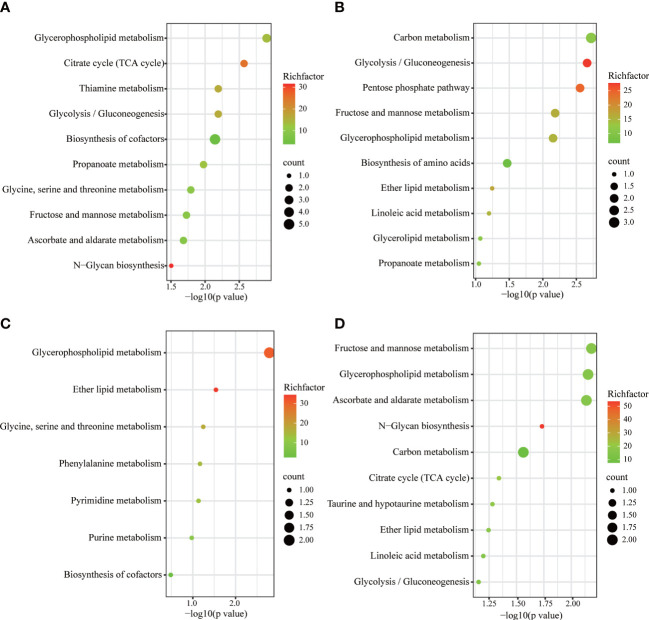
KEGG metabolic pathways enriched by different metabolites in hippocampus. **(A)** Control vs SD. **(B)** SD+FGL vs SD. **(C)** SD+FGH vs SD. **(D)** SD+EZ vs SD. The larger value of -log 10(*p* value) represents a more reliable metabolic pathway. The richfactor is positively correlated with the enrichment level. The size of the dots indicates the numbers of differentially expressed metabolites enriched.

Metabolic pathways were screened based on the number of differential metabolites, combined with log10 (*p* value) and rich factor. The horizontal coordinate -log(*p* value) is positively correlated with the degree of enrichment. The rich factor refers to the ratio of the number of metabolites enriched in the entry to the number of all metabolites annotated in the entry. The greater the enrichment factor, the higher the enrichment of differential metabolites.The KEGG was used to enrich relevant metabolic pathways (**Figure 7**). Metabolites which differed between the SD and control groups mainly involved the following metabolic pathways: glycerophospholipid metabolism, citrate cycle (TCA cycle), thiamine metabolism, and glycolysis/gluconeogenesis. Compared to the model group, the FGL majorly involves the following pathways: carbon metabolism, glycolysis/gluconeogenesis, pentose phosphate pathway, fructose and mannose metabolism, as well as glycerophospholipid metabolism. The metabolic pathways involved upon FGH treatment include glycerophospholipid metabolism, ether lipid metabolism, as well as glycine, serine and threonine metabolism. The metabolic pathways enriched in the SD+EZ group include fructose and mannose metabolism, glycerophospholipid metabolism, ascorbate and aldarate metabolism, among others.

### 3.4 Gut microbiota analysis

#### 3.4.1 Diversity analysis

**Figure 8:**
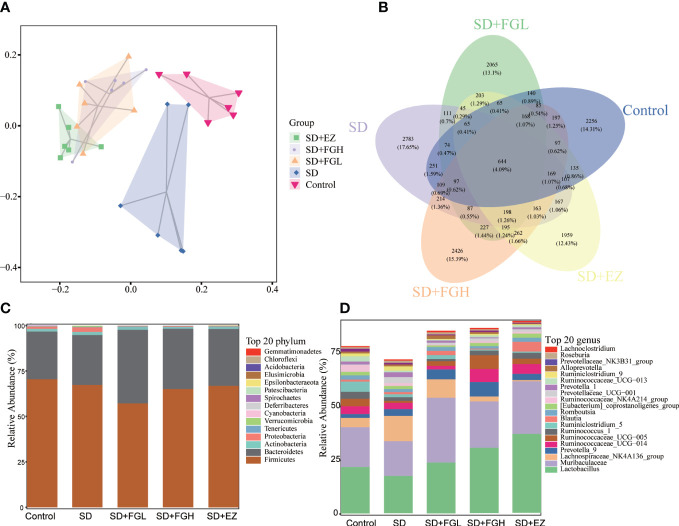
Effects of FG on the overall structure and composition of intestinal microflora in rats with SD-induced anxiety. **(A)** PCoA diagram showing changes in β- diversity. **(B)** Venn diagram of intestinal microbiota:the number of OTUs in intestinal contents. **(C)** Relative abundance of intestinal microbiota at the phylum level. **(D)** Relative abundance of intestinal microbiota at the genus level.

The richness and evenness of species within each community were assessed by α-diversity. Simpson and Shannon were used to reflect the diversity of the sample populations, and the observed species and Chao1 were used to assess the richness of the gut bacteria. There is no statistical significance in the overall difference of the five groups of samples (p>0.05) (**Supplementary Figure 4**). Based on the unweighted UniFrac PCA analysis of the differences in gut microbial community structure, as shown in **Figure 8A**, the control and SD groups were clearly separated in the PCA plots. This indicated that there were significant changes in gut microbiota between the control and SD groups. However, the sample distribution of PCA tended to be closer to the control group after FGL, FGH, and EZ intervention. This suggests that the treatment groups impacted the intestinal microbiota of the rats.

#### 3.4.2 Species composition structural analysis

To investigate the species composition of each sample, a veen plot visualizing the distribution of OTUs (**Figure 8B**) was constructed. The species shared by the Control, SD, SD+FGL, SD+FGH, and SD+EZ groups included 644 OTUs, with 2256 OTUs unique to the Control group, 2783 OTUs unique to the SD group, 2065 OTUs unique to the SD+FGL group, 2426 OTUs unique to the SD+FGH group, and 1959 OTUs unique to the SD+EZ group. These results were consistent with the alpha-diversity results. 16S rRNA sequencing demonstrated the results of species annotation analysis for each group at the phylum and genus levels. As shown (**Figures 8C, D**), the microbial community structure of each group at the phylum level saw over 90% of the total bacteria classified into the Firmicutes, Bacteroidetes, Actiobacteria, and Proteobacteria. Compared to the control group, the abundance of Firmicutes in the SD group had a slightly decreasing trend without statistical significance. Compared to the model group, the intervention group had a slight decrease in the abundance of Firmicutes and a slight increase in the abundance of Bacteroidetes. With respect to Proteobacteria, the abundance of SD group was higher than that of control group. However, after the intervention of FGL, FGH or EZ, the abundance of Proteobacteria tends to decline. The changes in intestinal microbiota were further characterized at the genus level. The relative abundance of *Lactobacillus* and *Muribaculaceae* both decreased and the relative abundance of *Lachnospiraceae_NK4A136_group* increased in the SD group compared to the control group. However, oral administration of FG or EZ reversed the trends observed in abundance of these microbiota.

#### 3.4.3 LEfSe analysis

**Figure 9:**
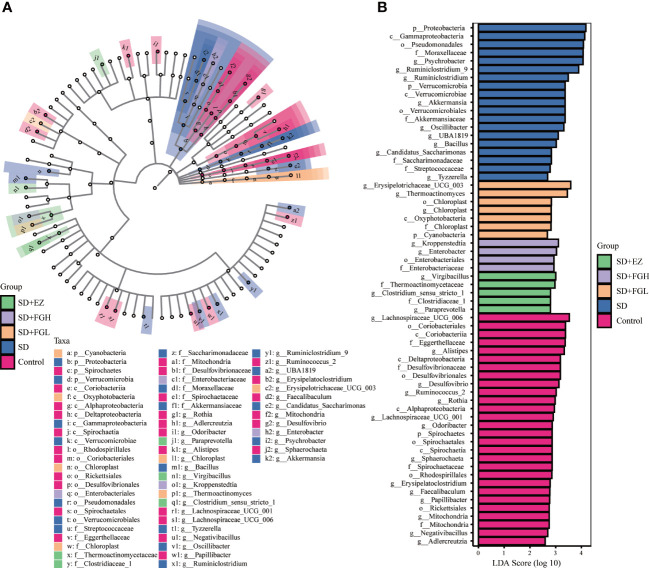
Relative abundance of intestinal microflora in rats with SD-induced anxiety. **(A)** Cladogram of bacterial taxa with different abundance. The hierarchical relationship of intestinal microflora in the sample community from Phylum to genus (from inner circle to outer circle). The node size corresponds to the average relative abundance of the taxon. **(B)** Histogram of LDA effect of different bacterial abundance. The ordinate is the intestinal microbiota with significant differences between groups, and the abscissa visually displays the logarithmic score of LDA analysis for each different microflora in the bar graph. The longer the length, the more significant the difference.

To identify the characteristic microbiota of rats associated with anxiety at the phylum and genus levels, the composition of the fecal microbiota of each group was compared using the LEfSe method with LDA values set to 2.0 and *p* < 0.05. As shown in **Figure 9**, the characteristic groupings observed within the control group at the genus included *Lachnospiraceae_UCG_006, Alistipes, Desulfovibrio, Ruminococcus_2, Rothia, Lachnospiraceae_UCG_001, Odoribacter, Sphaerochaeta, Erysipelatoclostridium, Faecalibaculum, Papillibacter, Negativibacillus*, and *Adlercreutzia.* However, the characteristic microbiota of the SD group of rats at the genus level were *Psychrobacter*, *Ruminiclostridium*_9, *Ruminiclostridium*, *Akkermansia*, *Oscillibacter*, *UBA1819, Bacillus*, *Candidatus_Saccharimonas*, and *Tyzzerella*. The FGL representative at the phylum level was *Cyanobacteria*, while at the genus level, the major characteristic groups were *Erysipelotrichaceae_UCG_003*, and *Thermoactinomyces*. At the genus level, representatives from FGH treatment *Kroppenstedtia* and *Enterobacter*. In the EZ group at the genus level, representatives included *Virgibacillus*, *Clostridium_sensu_stricto_1*, and *Paraprevotella*.

#### 3.4.4 Correlation analysis of differential hippocampal metabolites with differential fecal microbiota

**Figure 10:**
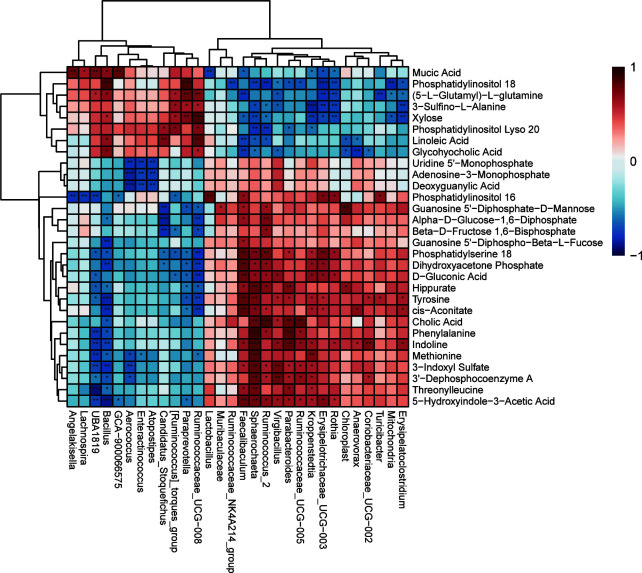
Heat map of the correlation between differential microbiota and changed hippocampal metabolites between the Control vs SD. Each row represents a differential hippocampal metabolite and each column represents a differential gut microbiota. Red indicates positive correlation; blue indicates negative correlation. ***p < 0.001, **p < 0.01, *p < 0.05 indicate statistically significant differences.

To investigate the functional correlation between metabolites in the hippocampus and gut microbe of anxious rats, correlation analysis was performed using Spearman’s correlation coefficient testing. As shown in **Figure 10** and **Supplementary Figure 5**, there were multiple associations between hippocampal metabolites and gut microbiota. *Muribaculaceae* in the SD group was positively associated with guanosine 5’−diphosphate−d−mannose (*p*<0.05). However, *Muribaculaceae* in the SD+FGL group was positively associated with Phosphatidylserine 18 (p<0.05), adenosine-3-monophosphate (p<0.05), dihydroxyacetone phosphate (p<0.05), and alpha−d−glucose−1,6−diphosphate (p<0.05). *Muribaculaceae* in the SD+FGL group was negatively correlated with Phosphatidylinositol 18 (p<0.05) and Phosphatidylinositol Lyso 20 (p<0.05). *Lactobacillus* in the SD group was positively associated with Phosphatidylinositol 16 (p<0.01), and negatively correlated with Mucic Acid (p<0.01). *Lactobacillus* in the SD+FGH group was positively associated with sn−glycero−3−phosphocholine (*p*<0.05), guanosine 5’−diphospho−beta−l−fucose (*p*<0.05), adenosine − 3−monophosphate (*p*<0.05), deoxyguanylic acid (*p*<0.05). This suggests that there may be a certain relationship between the metabolites of hippocampus and intestinal microbiota.

## 4 Discussion

Sleep is essential for mental and emotional stability. Sleep disorders may lead to mood disorders such as anxiety and depressive symptoms, as well as causing oxidative stress and inflammatory responses *in vivo* (Baum et al., 2014; Cox and Olatunji, 2016). In this study, we restricted the sleep time of rats through intraperitoneal injection of PCPA. After OFT and EPM behavioral tests, we established a rat model of SD-induced anxiety-like behavior. In addition, we observed significantly higher levels of the inflammatory factors TNF-α and IL-1β in the hippocampus of anxious rats compared to control group rats. However, like the standard drug EZ group, FGL and FGH demonstrated improved anxiety-like behavior and reduced levels of TNF-α and IL-1β in the hippocampus of SD rats 7 days after the intervention. To further explore the mechanism of FG efficacy, metabolomic analysis illustrated that FG could modulate disturbance of metabolites such as amino acids, lipids, and carbohydrates in the hippocampus of the model group, making the metabolic profile closer to the control group. Moreover, this analysis demonstrated that differential metabolites could be enriched in metabolic pathways such as carbon metabolism, glycolysis/gluconeogenesis pentose phosphate pathway, ether lipid metabolism, and glycine, serine, and threonine metabolism. Meanwhile, FG ameliorated disorders of intestinal microbiota in anxious rats and increased the abundance of beneficial bacteria such as *Lactobacillus*, *Muribaculaceae*.

OFT and EPM tests are widely used to evaluate mental behavior in experimental animals, including anxiety like behavior (Mishra et al., 2016). We found that the time and frequency of exploration of the central zone were reduced in the SD group of rats in the OFT compared to the normal group, and the OT% and OE% was also significantly reduced in the EPM. Previous studies have found that both acute and chronic SD cause animals to behave abnormally in both the OFT and EPM, accompanied by inflammation in the central nervous system (Manchanda et al., 2018; Kang et al., 2021). Glial cells are the immune cells of the central nervous system, and activation of glial cells increases levels of inflammatory factors including TNF-α, IL-1β, and IL-6 in the nervous system (Wadhwa et al., 2018). It was found that *Gardenia jasminoides* extract (GJ-4) could inhibit microglial activation and LPC-induced release of pro-inflammatory cytokines TNF-α and NO (Zang et al., 2022). Genipin, which is FG extract, reduced the expression of iNOS, COX-2, IL-1β, and IL-6 mRNA in the hypothalamus, amygdala, and BV-2 cells and ameliorated lipopolysaccharide-induced behavioral changes in animals with abnormal mood (Araki et al., 2014). Similar to these studies, we found that SD-induced abnormal emotional behavior was accompanied by an increase in the level of inflammation, while FG reduced the level of inflammation.

SD can impact certain lipid levels in organisms, and influence lipid metabolism. Acute sleep deprivation on plasma metabolite rhythms found that 24 lipid metabolites were significantly increased during sleep deprivation compared to during sleep periods (Davies et al., 2014). Phosphatidylinositol plays a very important role in cell morphology, metabolic regulation, signaling, and various physiological functions (Balla, 2013). In signaling pathways, phosphatidylinositol 4,5-bisphosphate (PIP2) is hydrolyzed into two messengers: inositol 1,4,5-trisphosphate (IP3) and diacylglycerol (DAG)(Chauhan-Puri et al., 2021). Research has demonstrated that the Ca^2+^-dependent signal mediated by the IP3 receptor is the basis of glial cell transmission and is a component of physiological processes such as sleep, neuroprotection, and learning memory (Chen et al., 2013; Nadjar et al., 2013). Extensive literature demonstrates that astrocytes regulate neuroinflammation, synaptic transmission, and plasticity through IP3 receptor-mediated Ca^2+^-dependent neurotransmitter molecules (Agulhon et al., 2012). In addition, protein kinase C (PKC) and long-term synaptic enhancement play key roles in the formation of reward memory and the reduction of anxiety-related behaviors. Inhibition of PKC activity attenuates the formation of reward memory and the alleviation of anxiety-related behaviors in mice (Lei et al., 2016). These studies suggest that FG may inhibit abnormal synaptic remodeling and anxiety-like behavior by interfering with the levels of phosphatidylinositol and impacting Ca^2+^ signaling and PKC activity.

Phosphatidylserine (PS) is a major acidic phospholipid, accounting for 13-15% of phospholipids in the human cerebral cortex. PS is involved in key signaling pathways in the nervous system, and unlike other membrane phospholipids, PS does not generate signaling through phospholipase-mediated hydrolysis to lead to the formation of bioactive products (Kim et al., 2010). Activation of Akt and PKC requires translocation from the cytoplasm to the plasma membrane, and this is critical for interaction with PS (Kim et al., 2014). Previous research found that phosphatidylserine has also been demonstrated to inhibit production of pro-inflammatory cytokines (Ma et al., 2011) and to promote anti-inflammatory pathways at the cellular level (Huynh et al., 2002). In our study, high and low dose of FG groups increased the level of PS metabolism, which may be the potential mechanism of its regulation of hippocampal inflammatory factors.

Glycerophosphocholine (GPC) is a water-soluble small molecule normally present in the body which provides choline necessary for the synthesis of acetylcholine and phospholipids. It is a biosynthetic precursor of acetylcholine. Acetylcholine is an important neurotransmitter in the central nervous system, helping the brain to regulate behaviors such as learning, memory, and anxiety (Zaborszky et al., 2018; Nakajima et al., 2020; Boiangiu et al., 2021). This function performed by choline in maintaining the structural integrity of membranes and regulating cholinergic neurotransmission may be dysregulated in certain neurodegenerative diseases (Bekdash, 2016). In addition, studies have found that dysregulated choline levels is associated with anxiety (Bjelland et al., 2009) and inflammation (Snider et al., 2018). In our study, we found that levels of glycerophosphorylcholine and choline in the hippocampus were disturbed after sleep deprivation. However, Gardenia jasminoides modulates the central choline disorder in rats.

Gluconic acid is produced enzymatically from glucose by activated glucose oxidase, which releases hydrogen peroxide. Therefore, gluconic acid can be considered as a marker of oxidative stress (Ament et al., 2021). SD has been shown to lead to oxidative damage, resulting in the production of reactive oxygen species (ROS), and the accumulation of ROS is a cellular threat that ultimately leads to cellular and neuronal damage (Salim, 2017). This oxidative damage hinders long-term potentiation (LTP) and leads to depression, anxiety, and impaired learning memory (Li et al., 2019). Research has shown that the increase of ROS in mice is related to anxiety behavior (Bouayed et al., 2009) and is also elevated in patients suffering from panic disorder, obsessive-compulsive disorder, and psychological distress states (Inoue et al., 2009; Miller and Sadeh, 2014). Numerous studies have illustrated that oxidative stress plays a pathogenic role in chronic inflammatory diseases (Miller et al., 2018; Steven et al., 2019). Excessive oxidative stress can lead to neuronal degeneration, and ROS produced in brain tissues alter synaptic and non-synaptic communication between neurons which leads to neuroinflammation and cell death (Kim et al., 2014).

Xylose, another important metabolite, is a monosaccharide with osmotic properties and is an intermediate product in sugar catabolism. Related studies show an association between xylose and inflammation levels (Komatsu et al., 2020). SD is also commonly associated with elevated levels of inflammatory proteins, an association that reflects impaired physiological functioning and the onset of the disease process (Irwin et al., 2010; Mullington et al., 2010). Acute sleep deprivation leads to activation of the hypothalamic-pituitary-adrenal (HPA) axis, which promotes secretion of pro-inflammatory cytokines, mediating the activation of astrocyte signaling, thereby triggering inflammatory activity and impaired neurological function in the central nervous system. These contribute to impacts on complex behavioral activities such as anxiety, cognition, appetite, and sleep (Sofroniew, 2014). The present study found that xylose levels were reduced after intervention with FGH and EZ compared to the model group.

Dihydroxyacetone phosphate and d-glucose-1,6-diphosphate are intermediate products in glycolysis. Glycolysis begins with glucose phosphorylation as the primary carbon source and is coupled to the TCA cycle to fully oxidize glucose and provide energy to the brain (Wang W. et al., 2020). Studies have demonstrated that anxious and depressed rats have highly active levels of glycolysis in the brain, but impaired tricarboxylic acid cycle, resulting in insufficient energy production (Detka et al., 2015; Jimenez et al., 2018). The reduced fructose 1,6-diphosphate levels observed in the hippocampus of SD rats may be due to reduced brain glucose uptake and changes in other glycolytic metabolites, suggesting that improved glycolysis may be the mechanism whereby the anxiolytic-like effects of FG are exerted.

The anabolism of amino acids is an important physiological activity in the human body, and metabolomic studies have identified a variety of amino acids involved in the regulation of human sleep rhythms (Hua et al., 2021). Betaine is an extremely important nutrient for the human body as it acts as a methyl donor for one-carbon metabolism. Betaine ameliorates oxidative stress and inflammation (Hagar et al., 2015), improves memory (Chai et al., 2013) and regulates emotion (Kim et al., 2013) *in vivo.* Betaine attenuates neurological damage in the hypothalamus of fructose-fed rats by inhibiting TLR4/NF-κB activation and HDAC3 overexpression to attenuate astrocyte proliferation and inflammatory responses (Li et al., 2015). In summary, betaine is associated with inflammatory and emotional centers of the central nervous system, and our study found that FGL increased betaine metabolism levels in the hippocampus.

Enrichment analysis of metabolic pathways demonstrated that most of the compounds differing between groups were found in glycerophospholipid metabolism, carbon metabolism, glycolysis/gluconeogenesis, pentose phosphate, and amino acid biosynthesis pathways. This suggests that lipid metabolism, gluconeogenesis and amino acid metabolism play important roles in the chemical changes within the hippocampus of SD-induced anxiety rats. Metabolomics have suggest that active ingredients rich in FG may inhibit SD-induced neuroinflammation, abnormal synaptic remodeling, and apoptosis in the hippocampus through alteration of metabolites in biological pathways such as lipid metabolism, glucose metabolism, and amino acid metabolism to maintain the structural and functional integrity of brain.

16S rRNA sequencing illustrated that at the phylum level, the two major phyla of Firmicutes and Bacteroidetes, changed their abundance and F/B ratio in association with insomnia and anxiety (Zhang et al., 2022a). Liang et al. found that the abundance of Bacteroidetes and Firmicutes, which dominate the mammalian intestinal microbiota, varied periodically from day to night (Liang et al., 2015). In this study, it was found that there were no significant changes in the Bacteroidetes and Firmicutes and their proportions in each group, and the reasons need to be further studied in the future. Proteobacteria are a major family of bacteria. All Gram-negative bacteria are Proteobacteria, including many pathogenic bacteria such as *Escherichia coli*, *Salmonella*, *Vibrio cholera*, and *Helicobacter pylori*. Proteobacteria have been defined as a major class of disease-causing bacteria, found to be associated with sleep and to production of endotoxins that promote chronic inflammation (Xiao et al., 2014; Wang Y. et al., 2020; Agrawal et al., 2021). Compared to normal sleepers, the number of Proteobacteria in short sleepers is enriched (Rizzatti et al., 2017; Zhang et al., 2017).

The FG group modulated intestinal microbiota in SD rats at the at the phylum level. Cyanobacteria work with other gut microbiota to ferment carbohydrates from food into short-chain fatty acids, which often peak at nightfall, a change that can be involved in regulating the circadian rhythm of sleep (Tahara et al., 2018). Moreover, melatonin may have evolved in Proteobacteria and in photosynthetic cyanobacteria. Melatonin plays a role in regulating sleep, circadian rhythms, enhancing immunity, and reducing oxidative stress through a receptor-mediated approach (Zhao et al., 2019). Receptor-specific ligand manipulation of 5-serotonin receptors(5-HT2C) by cyanobacteria alters anxiety and depressive behavior in mice (Lax et al., 2016). A study also uncovered that cyanobacterial extracts (DUQ0002I) containing serotonin receptor subtype 7 (5-HT7 R) reduced depression and anxiety-like behavior in mice (Lax et al., 2018).

At the genus level, *Lactobacillus* decreased in the sleep deprivation group compared to the control group, and this trend was reversed after pharmacological intervention. *Lactobacillus*, which belongs to a group of beneficial bacteria, reduces depression and anxiety, improves the intestinal microbial environment, increases the production of SCFAs, and reduces intestinal inflammation (Wei et al., 2019; Sovijit et al., 2021). In a previous study, SCFAs in feces of insomniac rats were reduced, and inflammatory factors were increased *in vivo* (Hua et al., 2021). SCFAs have been demonstrated to be key molecules connecting the intestine and brain, which can improve the inflammatory response of the central nervous system (Fung et al., 2017; Tankou et al., 2018; Tian et al., 2019). *Muribaculaceae* belong to the Bacteroides and are considered beneficial bacteria involved in biological processes such as energy, lipid, and glucose metabolism. *Muribaculaceae* were found to be reduced in abundance in both anxiety patients and rat models (Chen Y. H. et al., 2019; Zhang et al., 2022a). As with *Lactobacillus*, *Muribaculaceae* also produce short-chain fatty acids that exert anti-inflammatory and neurotransmitter-regulating effects (Smith et al., 2019). *Lachnospiraceae NK4A136* has been associated with vestibular disorders and neuropathic pain combined with anxiety (Li et al., 2022; Shen et al., 2022). Furthermore, in a mouse model of colitis, the abundance of the *Lachnospiraceae NK4A136* was increased, suggesting that *Lachnospiraceae NK4A136* may be associated with anxiety and inflammation(Shao et al., 2020). *Clostridium_ sensu_ Stricto_ 1* is a characteristic bacterial group in the EZ group, belonging to Clostridium, most of which can produce butyric acid. Butyric acid has important metabolic functions which are essential for various physiological processes, including maintenance of intestinal barrier function, inhibition of systemic inflammation, and regulation of endocrine metabolism levels (Simon et al., 2020). No depression and anxiety-like behaviors were observed in obesity-resistant mice, and higher abundance of *Clostridium_sensu_stricto_1* was observed *in vivo* (Pang et al., 2022). In conclusion, FG was responsible for regulating the intestinal bacteria of anxiety model rats, increasing the abundance of beneficial bacteria which can then produce short-chain fatty acids, regulate neuroinflammation and neurotransmitters, and exert anxiolytic and sleep-regulating effects. The positive drug EZ also impacted the intestinal microbiota, but whether the mechanism is related to its sedative and hypnotic effects requires confirmation in subsequent related experiments.

The intestinal microbiota and metabolism within the central nervous system are correlated. Gut microbiota can produce metabolites such as SCFAs, amino acids, and indole derivatives which impact CNS metabolism and function through neural, endocrine, and immune pathways (Collins et al., 2012). Metabolomics reveals that SD-induced anxiety models result in disturbances in hippocampal metabolism of amino acids, lipids, and carbohydrates, etc (Si et al., 2022). Spearman’s correlation analysis revealed that presence of *Muribaculaceae* is positively correlated with phosphatidylserine 18, which significantly decreases IL-1β production and increases IL-10 levels *in vivo* (Ma et al., 2011). The significant different microbiota *Ruminiclostridium* in the model group was negatively correlated with betaine, which has anti-inflammatory, anti-apoptotic, and anti-depressant effects (Kim et al., 2013; Hagar et al., 2015). In summary, correlation analysis of gut microbiota and metabolomics demonstrated that anxiety, inflammation, gut microbiota, and hippocampal metabolism are correlated, confirming the existence of a link between microbial communities and the brain.

Although our study confirmed the anxiolytic effect and mechanism of FG, shortcomings still remain. The present study confirms that SD induces anxiety-like behaviors accompanied by neuroinflammation in rats, but the pathways upstream of inflammation, including genetics and activation of glial cells have not been experimentally studied. Disturbances in hippocampal metabolic profiles are involved in the pathological process of SD-induced anxiety. We maintained objectivity and accuracy in identifying hippocampal metabolites and screening for endogenous differential metabolites, but future experimental validation is still required. The determination of SCFAs and the study of serum and plasma metabolomics can help to fully understand the relationship between the gut microbiota and the central nervous system. To some extent, this study confirmed the correlation between intestinal bacteria, hippocampal metabolism, inflammation, and anxiety like behavior. In the future, fecal microbiota transplantation (FMT) will be required to confirm the causal relationship between phenotype and mechanism. Finally, this study was conducted only on FG, and further screening of the active ingredients of FG for anxiolysis will be needed.

In summary, FG improved SD-induced anxiety in rats. FG reduced neuroinflammation in the hippocampus and modulated phosphatidylserine 18, sn-glycero-3-phosphocholine, deoxyguanylic acid, d-gluconic acid and other metabolites. Differential hippocampal metabolites are involved in the main metabolic pathways: carbon metabolism, glycolysis/gluconeogenesis, pentose phosphate pathway and glycerophospholipid metabolism. Meanwhile, FG mainly increased the abundance of the intestinal microbiota *Muribaculaceae, Lactobacillus* and decreased the abundance of *Lachnospiraceae_NK4A136_group*. In conclusion, FG exerts anxiolytic effects through the regulation of neuroinflammation, hippocampal metabolites and intestinal microbiota.

## Data availability statement

The datasets presented in this study can be found in online repositories. The names of the repository/repositories and accession number(s) can be found below: https://www.ncbi.nlm.nih.gov/, PRJNA937043.

## Ethics statement

The animal study was reviewed and approved by Animal research by animal review and approval of the Ethics Committee of Hebei University of Chinese Medicine.

## Author contributions

DL, JM conceived the original idea and designed the experiments; DL, QW, YL analyzed the data and wrote the manuscript; ZY contributed to the discussion section of the manuscript; DL, QW, ZL, JG, XL, WZ, YT performed the animal experiments; JM reviewed and revised the manuscript; all authors approved the submission of the manuscript.
