# Supplementary material for: Fructus gardeniae ameliorates anxiety-like behaviors induced by sleep deprivation via regulating hippocampal metabolomics and gut microbiota
Source: Front Cell Infect Microbiol. 2023 Jun 12;13:1167312. doi: 10.3389/fcimb.2023.1167312 (PMC10291143; doi:10.3389/fcimb.2023.1167312)
Supplement: Supplementary file 1 [file DataSheet_1.zip › Supplementary documents and pictures6.4.docx]

Supplementary Material

**Fructus gardeniae ameliorates anxiety-like behaviors induced by sleep deprivation via regulating hippocampal metabolomics and gut microbiota**

Dong Liu^1,2^, Qianfei Wang^1^, Ying Li^3^, Zhenshuang Yuan^4^, Zhiliang Liu^5^, Junli Guo^1^, Xin Li^1^, Weichao Zhang^1^, Yulei Tao^1^, Jianqiang Mei^1*^

^1^ Department of Emergency, The First Affiliated Hospital of Hebei University of Chinese Medicine, Shijiazhuang, Hebei, China, ^2^ Department of Traditional Chinese Medicine, Hebei General Hospital, Shijiazhuang, Hebei, China, ^3^ Department of Pharmacy, The First Affiliated Hospital, and College of Clinical Medicine of Henan University of Science and Technology, Luoyang, China, ^4^ School of Chinese Materia Medica, Beijing University of Chinese Medicine, Beijing, China ^5^ Department of Emergency, Hebei Yiling Hospital, Shijiazhang, Hebei, China.

***correspondence:**Jianqiang Mei

1. mail: mjq1000000@sina.com


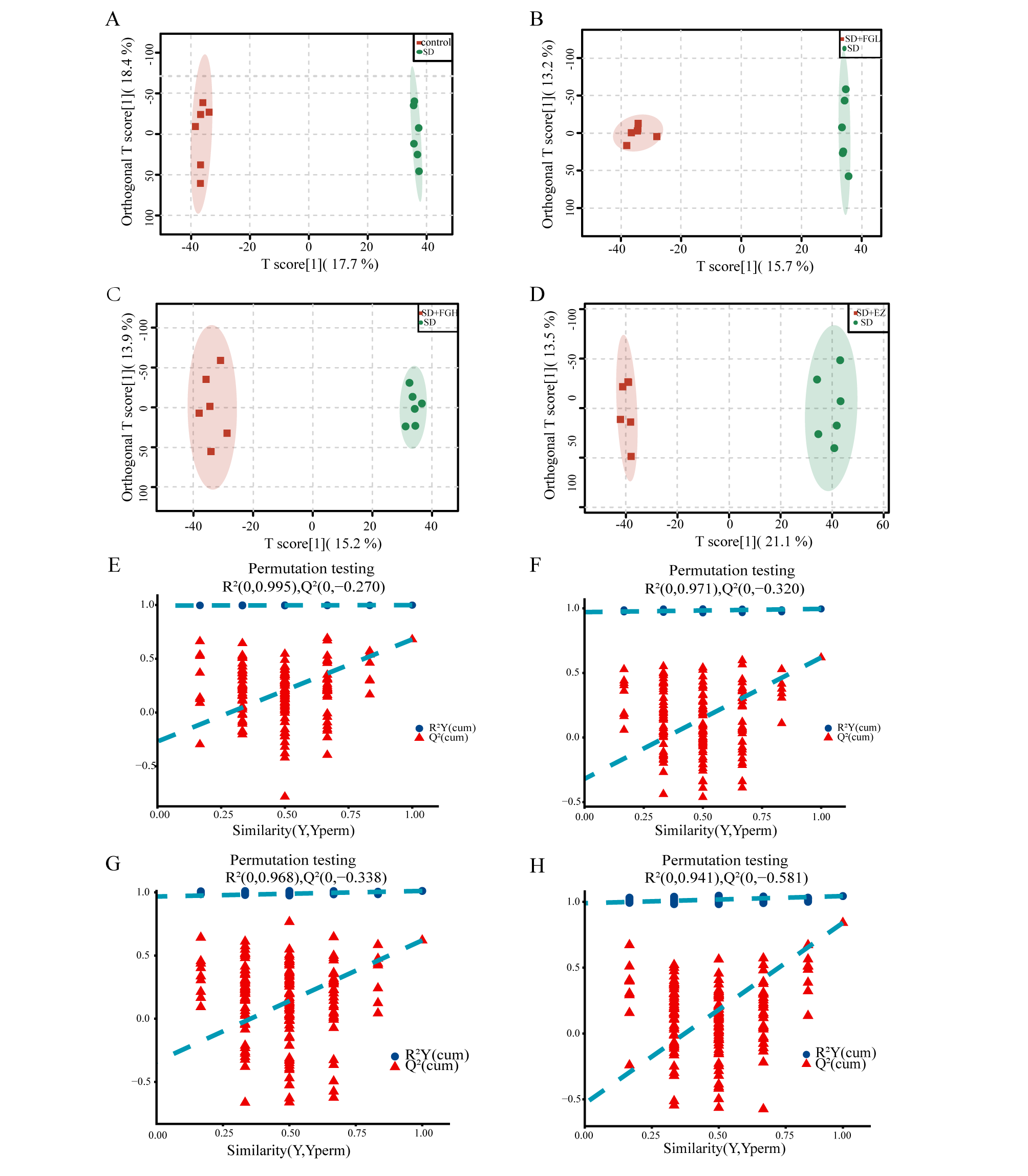
**Supplementary Figure 1** OPLS-DA statistical analysis results and model stability verification. (A-D) OPLS-DA analysis results in negative mode. (A) Control vs SD (R^2^Y=0.999 , Q^2^=0.680). (B) SD+FGL vs SD (R^2^Y=0.996, Q^2^=0.619 ). (C) SD+FGH vs SD (R^2^Y=0.995, Q^2^=0.604). (D) SD+EZ vs SD (R^2^Y=0.995, Q^2^=0.792); (E-H) Permutation experiment results in negative mode. (E) Control vs SD. (F) SD+FGL vs SD. (G) SD+FGH vs SD. (H) SD+EZ vs SD.


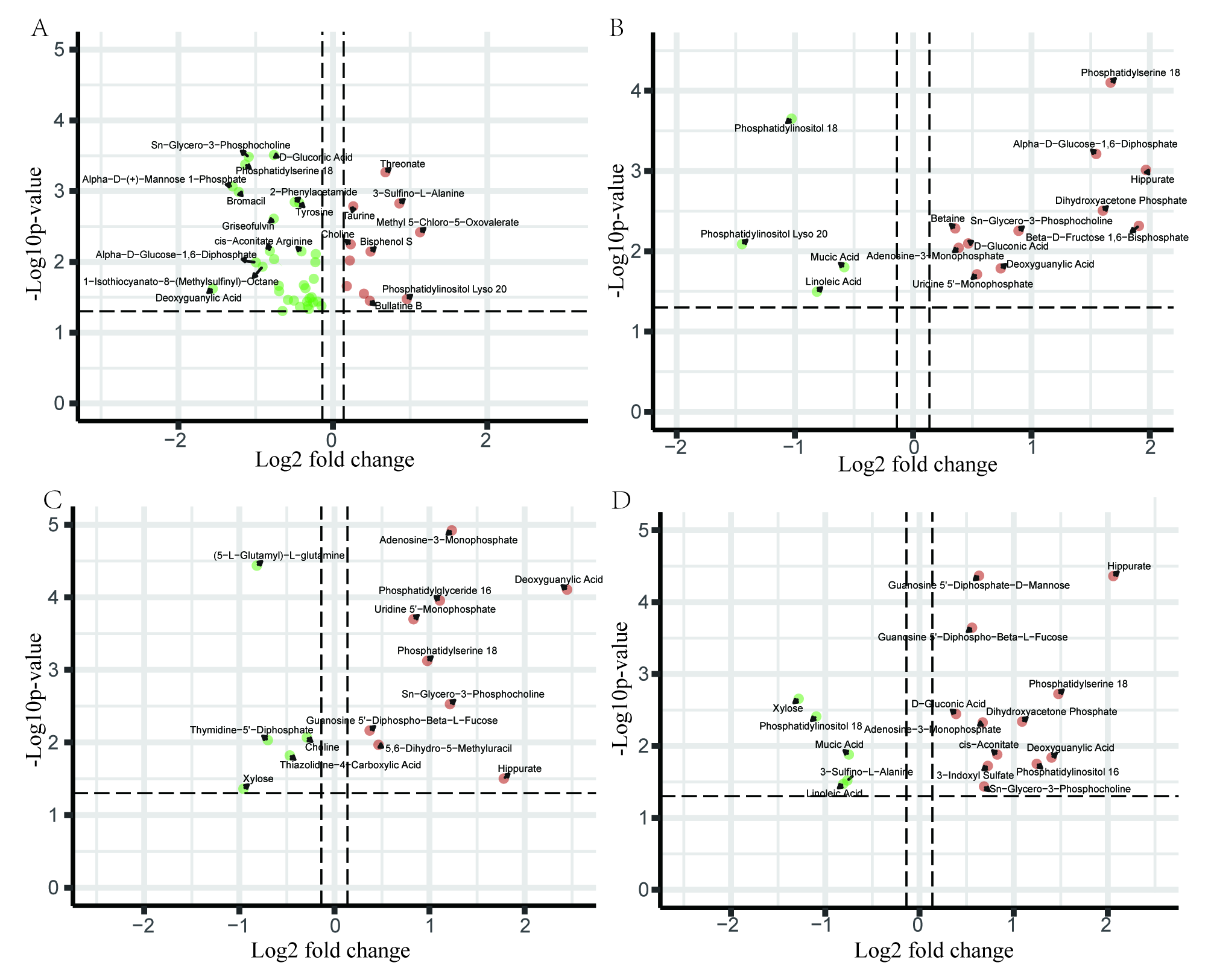


**Supplementary Figure 2** Volcano map of gene differential expression between two samples. A: Control vs SD. B. SD+FGLvs SD. C:SD+FGH vs SD. D: SD+EZ vs SD. The X-axis log2FoldChange indicates the degree of difference. The Y-axis - Log10*P*-value indicates significance. The red dot represents the up-regulated metabolites, and the green dot represents the down-regulated metabolites.


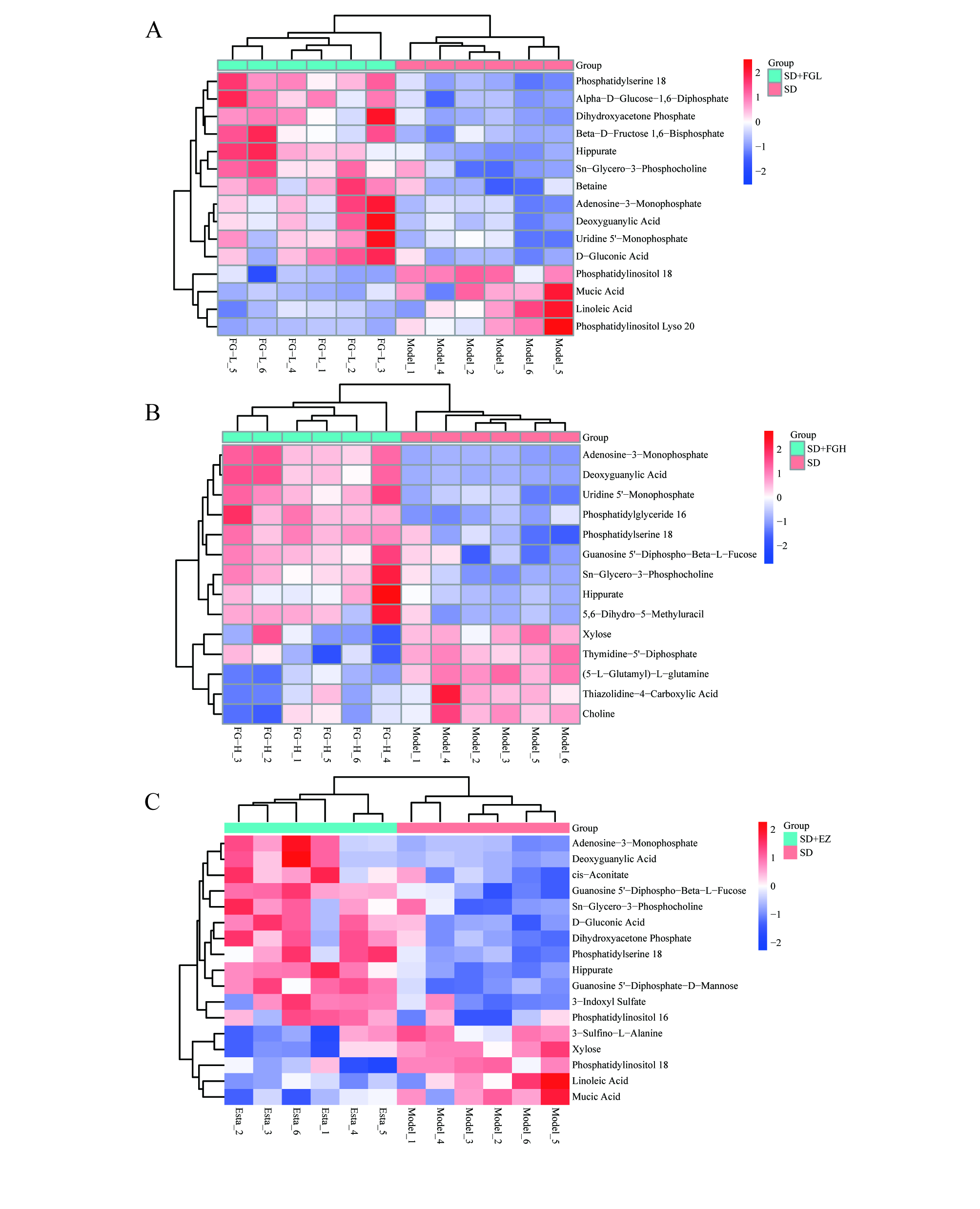
**Supplementary Figure 3** Heat map of different metabolite levels in hippocampus. A: SD+FGLvs SD. B:SD+FGH vs SD. C: SD+EZ vs SD. The abscissa represents the sample, and the ordinate represents the metabolites. Red and blue represent up-regulation and down-regulation of hippocampal metabolites, respectively.

**
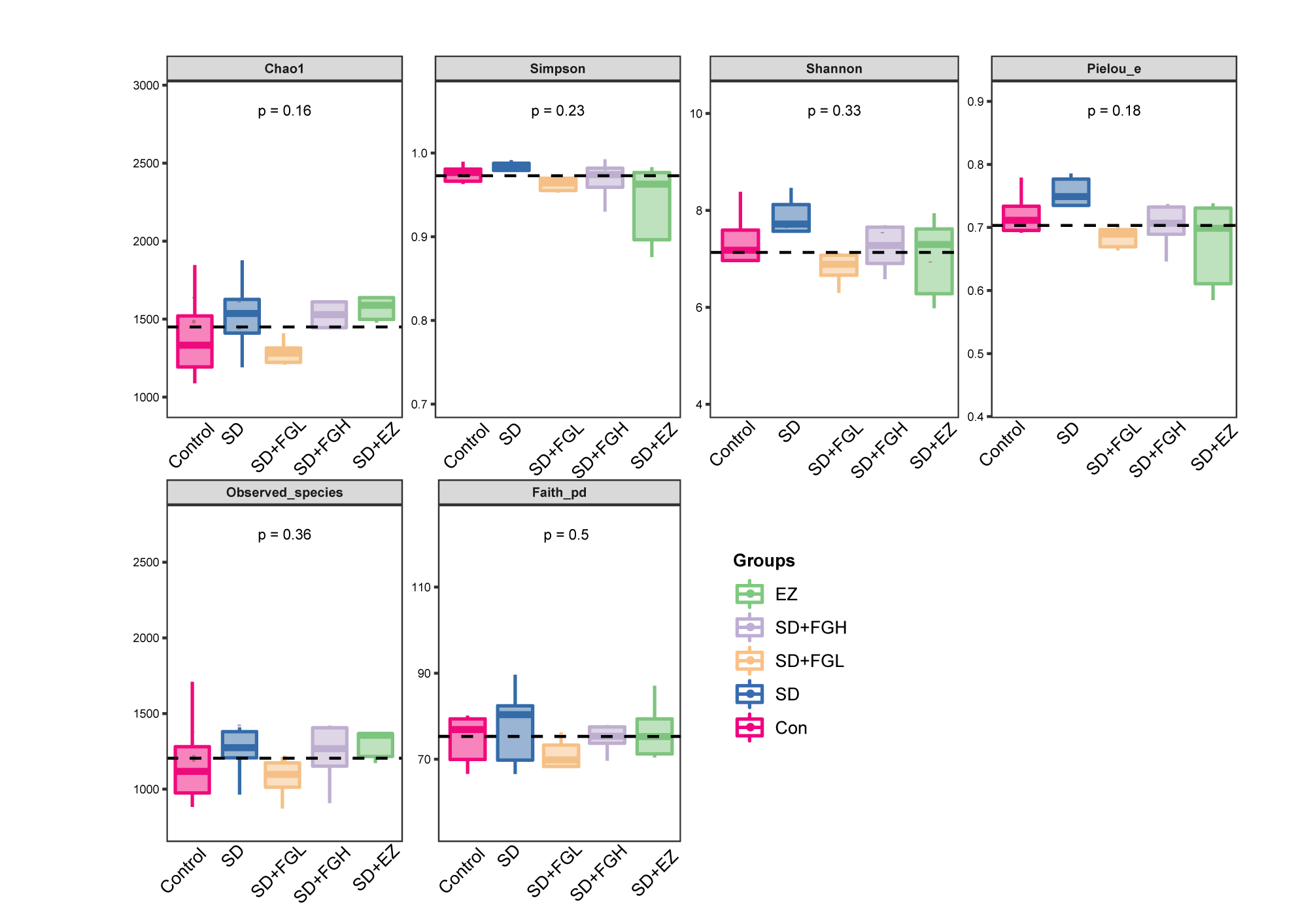
Supplementary Figure 4 :** The α-Diversity of intestinal microbiota. There is no statistical significance in the overall difference of the five groups of samples (p＞0.05).


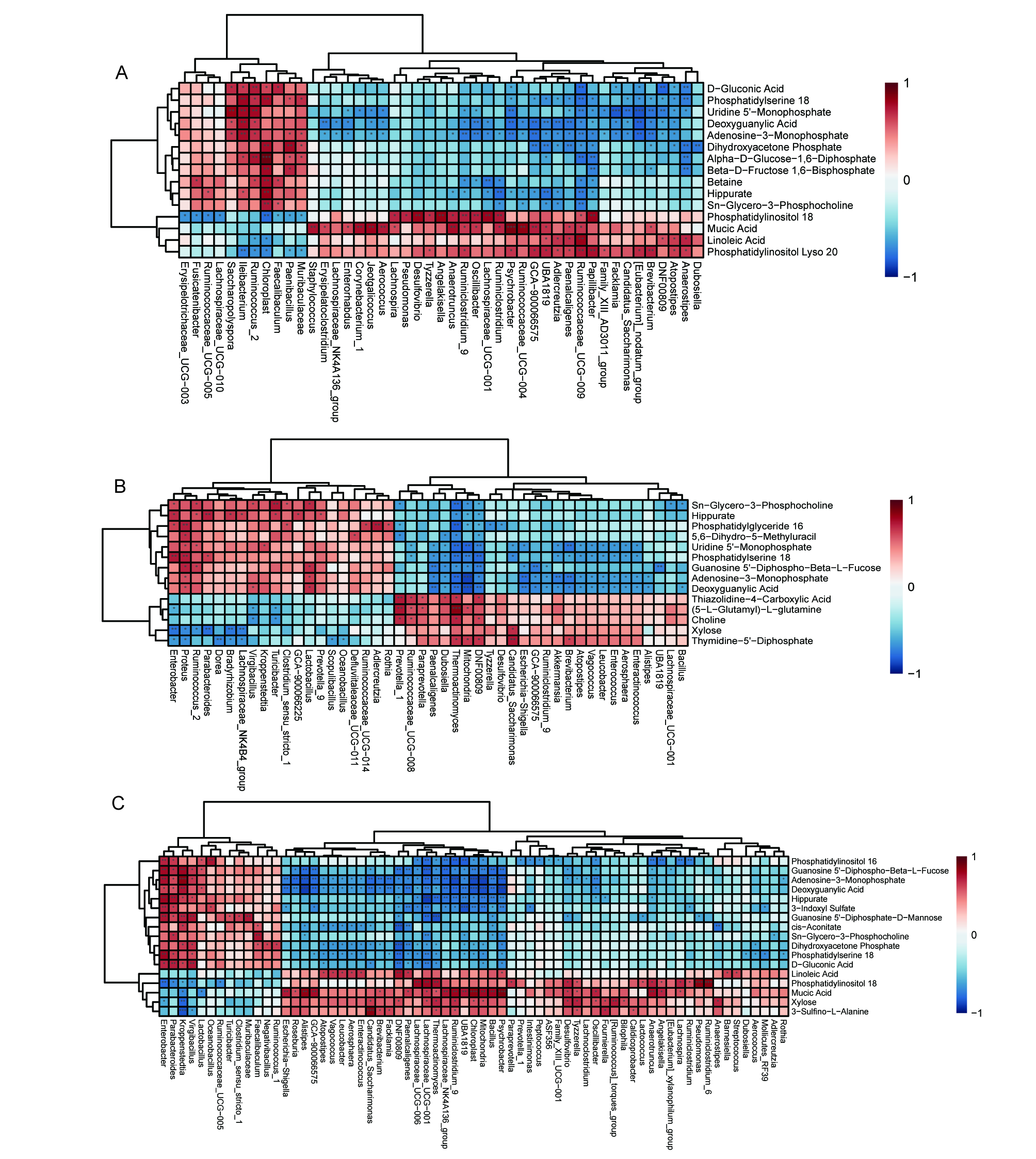


**Supplementary Figure 5** Hierarchical clustering heat map of the correlation between differential microbiota and changed hippocampal metabolites. (A) SD+FGL vs SD. (B) SD+FGH vs SD. (C) SD+EZ vs SD. Red indicates positive correlation, blue indicates negative correlation. ****p* < 0.001, ***p* < 0.01,* *p* < 0.05 indicates statistically significant differences.
